# A Point Temperature Sensor Based on Upconversion Emission in Er^3+^/Yb^3+^ Codoped Tellurite-Zinc-Niobium Glass

**DOI:** 10.3390/s17061253

**Published:** 2017-05-31

**Authors:** Ting Wu, Rui Tong, Liwen Liao, Lihui Huang, Shilong Zhao, Shiqing Xu

**Affiliations:** College of Materials Science and Engineering, China Jiliang University, Hangzhou 310018, China; S1505080518@stu.cjlu.edu.cn (T.W.); S1405080511@stu.cjlu.edu.cn (R.T.); 1400503218@stu.cjlu.edu.cn (L.L.); huanglihui@cjlu.edu.cn (L.H.); shiqingxu@cjlu.edu.cn (S.X.)

**Keywords:** temperature sensing, upconversion emission, tellurite-zinc-niobium glass

## Abstract

Er^3+^/Yb^3+^ codoped tellurite-zinc-niobium (TZNb) glass was prepared by the melt-quenching method and used for the construction of a point all-fiber temperature sensor. The glass thermal stability and network structural properties were studied by differential thermal analysis and Raman spectrum, respectively. High glass transition temperature is beneficial to widen the working temperature range. The dependence of fluorescence intensity ratio (FIR) of green upconversion emissions on the surrounding temperature from 276 to 363 K was experimentally investigated and the maximum temperature sensitivity is 95 × 10^−4^ K^−1^ at 363 K. Strong green upconversion emission, broad temperature measurement range and high sensitivity indicate this point temperature sensor is a promising optical device for application on optical temperature sensing.

## 1. Introduction

Temperature is one of the most fundamental physical parameters and accurate measurement of temperature plays an important role in industrial production and scientific research. In contrast to conventional measurement methods, as a kind of non-contact temperature measurement method, optical fiber temperature sensors have the advantages of high accuracy, dynamic response, corrosion resistance, immunity to electromagnetic fields and are suited for the temperature measurements of harsh environments (such as high current and high magnetic field, etc.) [1,2,3]. Compared with bulk sensors, the advantages of optical fiber temperature sensors are obvious. For example, the small sensor probe makes it easy to detect in a small space. The optical fiber temperature sensor based on fluorescence intensity ratio (FIR) technique was reported by H. Bertthou for the first time and had attracted great attention because of its simple structure, convenient data processing, and high sensitivity [4,5]. FIR technique is based on the large variation of the relative fluorescence intensities from two thermally-coupled levels of rare earth ions, when the environmental temperature changes. By establishing the relationship between FIR and temperature, temperature could be easily determined. 

In order to improve the sensitivity and broaden temperature measurement range of optical temperature sensors, the design of a sensor probe is the focus of the research. On the one hand, the strong luminescence intensity can reduce the excitation power and improve the temperature sensitivity; on the other hand, the high operating temperature is helpful to widen the measurement range of the temperature sensor [6]. Due to its low phonon energy and broad transmission range from ultraviolet to mid-infrared, fluoride glass is the first choice. However, the poor chemical stability and low glass transition temperature limit its potential application on the optical fiber temperature sensing. In comparison to silicate (1100 cm^−1^) [7] and phosphate glasses (1300 cm^−1^) [8], tellurite glasses possess the relatively low phonon energy (750 cm^−1^) [9], high thermal stability, and high solubility of rare earth ions and are usually used for host matrix. By now, the temperature sensing characteristic of Er^3+^ single doped and Er^3+^/Yb^3+^ codoped tellurite glasses have been investigated. In 2010, the temperature dependence of upconversion luminescence in Er^3+^/Yb^3+^ codopedtellurite glasses was reported [10]. In 2013, the effect of Er^3+^ concentration on the temperature-dependent upconversion emission in fluorotellurite glasses were investigated and the maximum thermal sensitivity was found to be 79 × 10^‒4^ K^‒1^ at 541 K for the fluorotellurite glass doped with the lowest concentration of Er^3+^ [11]. In 2014, the visible upconversion and temperature sensing behavior of Er^3+^-Yb^3+^ doped/codoped TeO_2_-WO_3_ glasses were researched and the temperature sensing performance has been studied by FIR technique up to 745 K, which effectively broaden the working temperature range of temperature sensor [12]. In the same year, a point temperature sensor based on Er^3+^/Yb^3+^ codoped tellurite glasses was developed and the dip coating technique was used to fabricate the optical fiber temperature sensing probe [13]. The temperature sensing characteristic was investigated at the range of 296–312 K and the maximum temperature sensitivity was 39 × 10^−4^ K^−1^.

By now, although a great deal of research work has focused on the development of rare earth doped optical materials, the construction of a prototype sensor is relatively lacking and the performance of optical temperature sensors needs further research [14]. Moreover, an all-fiber temperature sensor is preferred for practical applications with the advantages of a compact structure, high sensitivity, and cost-effectiveness. In this paper, the thermal stability, glass network structure and upconversion emission of Er^3+^/Yb^3+^ codoped TZNb glass were investigated. A point temperature sensor with all-fiber structure was designed and the performance of temperature sensing was evaluated by exploring the change of FIR dependent temperature from 276 K to 363 K, and the maximum temperature sensitivity is 95 × 10^−4^ K^−1^ at 363 K.

## 2. Experimental Section

Er^3+^/Yb^3+^ codoped TZNb glass was prepared by the traditional high temperature melt quenching method. The molar composition of TZNb glass is 80TeO_2_-10ZnO-10Nb_2_O_5_, with the addition of 0.25 mol % Er^3+^ and 0.5 mol % Yb^3+^ ions. All raw materials were completely mixed and melted in a corundum crucible at 1173 K for 30 min in order to get a uniform glass liquid. Then, the melt was poured into a preheated brass plate. Subsequently, TZNb glass was rapidly transferred to muffle furnace and annealed at 673 K for 2 h in order to release the residual stress. 

The glass density was measured by Archimedes method. Refractive index was determined on the Mectricon Models 2010/M prism coupler and laser wavelength of 632.8 nm. The glass transition temperature and onset crystallization temperature were taken on the Netzsch DTA404PC. X-ray diffraction measurement was determined onthe Bruker D2 PHASER Diffractometer. Raman spectrum was measured by RenishawInvia confocal Raman spectrometer. The room temperature upconversion luminescence was measured by the Jobin-YvonFrolog 3 fluorescence spectrometer, and the excitation wavelength was 980 nm. 

**Figure 1:**
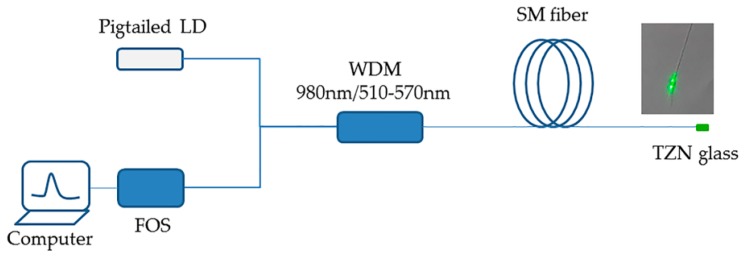
Schematic diagram of experimental arrangement used for optical fiber temperature sensing. The upper right corner presents the strong green upconversion emission at a power of 1 mW.

A all-fiber point temperature sensor was developed and the corresponding experimental arrangement is shown in the Figure 1. A single mode pigtailed laser diode at 976 nm was used to excite the temperature probe and its power could be regulated by a current controller. After passing through a wavelength division multiplexer (WDM, 980 nm/510–570 nm), the pumping beam enters single mode (SM) silica fiber (Corning Hi1060 specialty fiber) and subsequently excites the temperature probe. The counter-propagating green beam was detected by a miniature fiber optic spectrometer (FOS) equipped with a SONY ILX554B CCD. The temperature probe was prepared by the fiber dip coating technique. First, the surface protective layer on the tip of SM silica fiber was removed, and then the fiber tip was rapidly immersed in the molten Er^3+^/Yb^3+^ doped TZNb glasses. Thus, a thin layer of TZNb glass on the end face of the SM fiber was formed and used for temperature sensing. The temperature of fiber probe was controlled at the range of 276–363 K. 

## 3. Results and Discussion

Table 1 shows the basic physical parameters of Er^3+^/Yb^3+^ doped TZNb glasses. Obviously, the glass transition temperature (*T_g_*) and onset crystallization temperature (*T_x_*) are located at 683 K and 875 K, respectively. Thus, the difference Δ*T* between *T_x_* and *T_g_* (Δ*T = T_x_* − *T_g_*) is 188 K. Generally, the difference ∆*T* is used as a rough criterion to evaluate the glass thermal stability and the desirable ∆*T* is believed to be more than 100 K [15]. This result suggests TZNb glass is characterized by high glass thermal stability. Furthermore, high glass transition temperature is very advantageous to enlarge the temperature measurement range of optical fiber temperature sensor.

**Figure 2:**
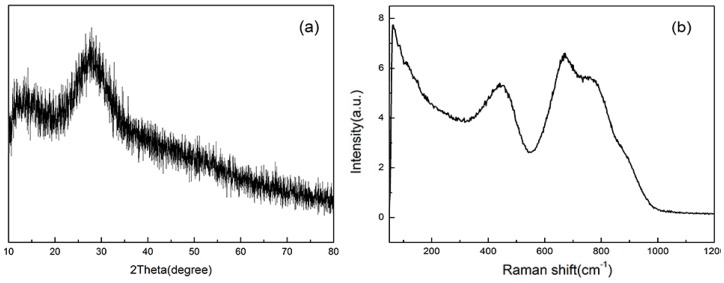
(**a**) The XRD pattern of TZNb glass; (**b**) Raman spectrum of undoped TZNb glass.

The X-ray diffraction result of TZNb is shown in the Figure 2a. The absence of sharp crystalline peaks and two broad humps confirm the amorphous nature. Raman spectrum was carried out to investigate the glass network structure of undoped TZNb glass and shown in Figure 2b. The asymmetric Raman peak located at 66 cm^–1^ is one of the characteristic peaks of Raman spectra of glass matrix [16]. The Raman peak centered at 431 cm^−1^ is attributed to the symmetric bending vibration of the Te-O-Te bonds. The strong and broad vibrations bands at 673 cm^–1^ and 756 cm^–1^ are derived from the stretching vibrations of [TeO_4_] trigonal bipyramids with bridging oxygen atoms and the stretching vibrations of [TeO_3+1_] and [TeO_3_] pyramidal units associated with non-bridging oxygen (NBO) units, respectively [17]. The weak Raman peak at 880 cm^–1^ originates from the vibration of Nb-O bond in the NbO_6_ unit [18]. Therefore, the maximum phonon energy of undoped TZNb glass is 880 cm^–1^. The relative low phonon energy is advantageous to enhance the upconversion luminescence intensity of rare earth ions.

**Figure 3:**
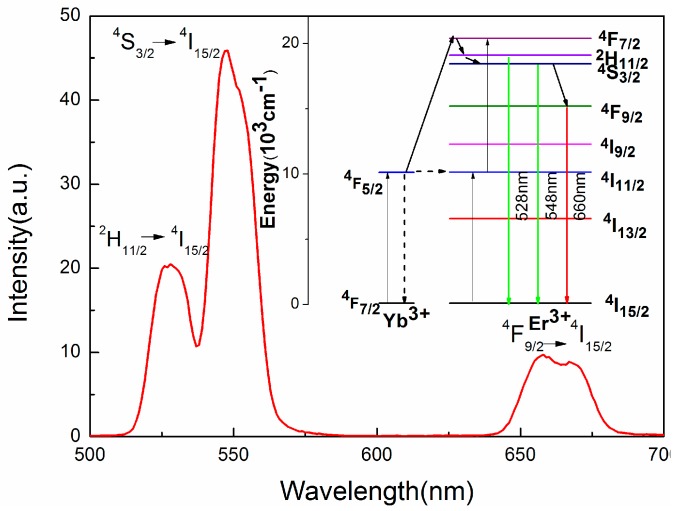
Upconversion luminescence of Er^3+^/Yb^3+^ doped TZNb glass. The inset shows the simple energy levels of Er^3+^ and Yb^3+^ ions and the possible upconversion mechanism in TZNb glass.

The upconversion luminescence of bulk Er^3+^/Yb^3+^ doped TZNb glasses at room temperature is shown in the Figure 3 and the excitation power of 980 nm semiconductor laser diode is 100 mW. Three major emission bands located at 528, 548, and 660 nm are observed, which could be easily assigned to the ^2^H_11/2_→^4^I_15/2_, ^4^S_3/2_→^4^I_15/2_, ^4^F_9/2_→^4^I_15/2_ of Er^3+^ ions, respectively. The inset of Figure 3 displays the simple energy levels of Er^3+^ and Yb^3+^ ions and the possible upconversion mechanism in the TZNb glass. Firstly, the population of excited level ^4^I_11/2_ of Er^3+^ ions may be populated by direct absorption a 980 nm photon or energy transfer from excited level ^2^F_5/2_ of Yb^3+^ ions. Because the absorption cross-section at 980 nm of Yb^3+^ ions is much larger than that of Er^3+^ ions, 980 photons are mainly absorbed by Yb^3+^ ions. Thus, the energy transfer process is predominant. Then, the metastable Er^3+^ ions at ^4^I_11/2_ level further absorb a second 980 nm photon or the energy transferring from excited level ^2^F_5/2_ of Yb^3+^ ions and are excited to ^4^F_7/2_ level. Subsequent nonradiative relaxation process ^4^F_7/2_→^2^H_11/2_, ^4^S_3/2_ and ^4^F_9/2_ leads to the population of ^2^H_11/2_, ^4^S_3/2_ and ^4^F_9/2_ energy levels, which produce strong green upconversion emission at 528 and 548 nm and weak red upconversion emission at 660 nm by radiative transition to the ground state, respectively. 

The temperature sensing behavior of the point optical fiber temperature sensor was studied by recording the upconversion emission spectra at the range of 276 to 363 K. The excitation power is fixed at 1 mW and strong green upconversion emission of Er^3+^ ions from ^2^H_11/2_ and ^4^S_3/2_ to ground state could be easily observed by the naked eye, as shown in the inset of Figure 1.

**Figure 4:**
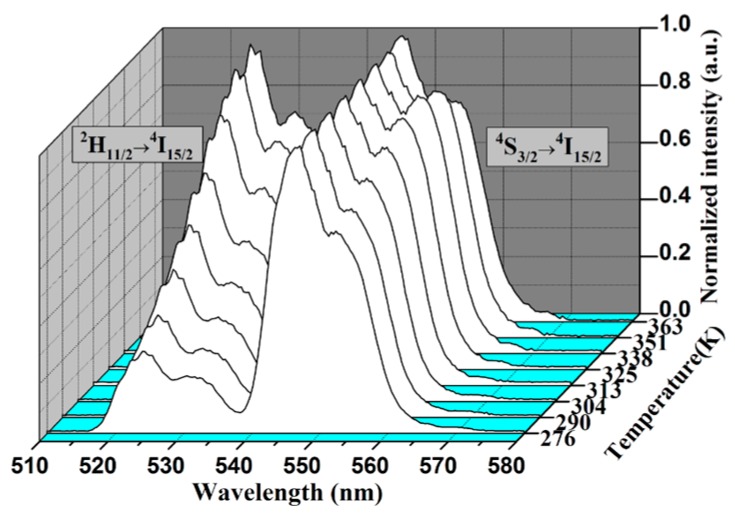
Effect of temperature on upconversion emissions in the Er^3+^/Yb^3+^ doped TZNb glass.

With the increase of temperature from 276 to 363 K, the relative fluorescence intensity ratio of two green upconversion emission bands changes dramatically while the peak positions do not change, as shown in the Figure 4. All the emission bands are normalized to the maximum intensity of ^4^S_3/2_→^4^I_15/2_ bands at each temperature. It is clear that the emission intensity of ^2^H_11/2_ level gradually increases, which is due to the increase of the population of ^2^H_11/2_ level at the expense of that of ^4^S_3/2_ level.

**Figure 5:**
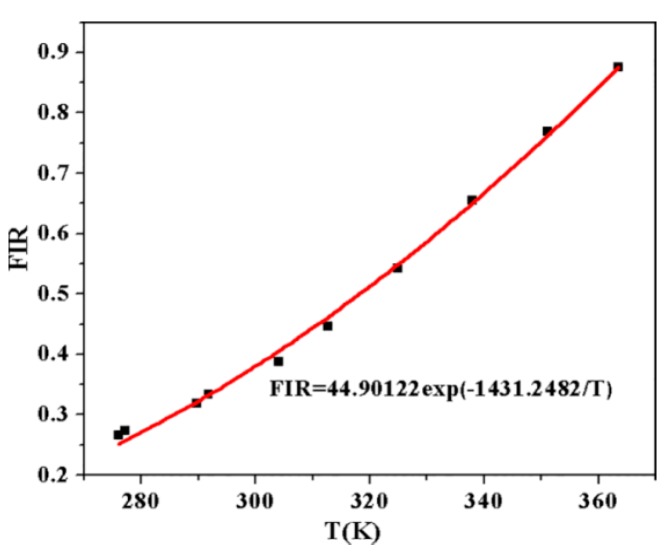
FIR of upconversion emission band as a function of temperature ranging from276 to 363 K.

As ^2^H_11/2_ and ^4^S_3/2_ are thermally coupled and their populations are in accordance with the Boltzmann distribution, the FIR of these two green upconversion emissions can be described as [19]:

(1) $$FIR=\frac{I_{H}}{I_{S}}=\frac{N{(}^{2}H_{11/2})}{N{(}^{4}S_{3/2})}=\frac{g_{H}\sigma_{H}\omega_{H}}{g_{S}\sigma_{S}\omega_{S}}\exp(\frac{-\Delta E}{kT})=C\exp(\frac{-\Delta E}{kT})$$

in which *I_H_* and *I_S_* are the integrated intensities corresponding to ^2^H_11/2_→^4^I_15/2_ and ^4^S_3/2_→^4^I_15/2_ transitions, respectively. *N, g, σ, ω* are the number of ions, the degeneracy, the emission cross-section, the angular frequency of fluorescence transitions from ^2^H_11/2_ and ^4^S_3/2_ levels to ^4^I_15/2_ level, respectively. ∆*E* is the energy gap between the thermally coupled levels ^2^H_11/2_ and ^4^S_3/2_, *k* = 0.695 cm^–1^/K is the Boltzmann constant. The FIR of 528 nm and 548 nm upconversion emission band as a function of temperature is shown in the Figure 5. FIR varies from 0.27 to 0.88 at the range of 276 to 363 K and the fitting values of *C* and Δ*E/k* are 44.90 and 1431, respectively.

To better understand the temperature sensing behavior, the investigation of the sensor sensitivity *S* is of great importance. Herein, *S* is defined as the rate at which the FIR varies with the temperature and is expressed as

(2) $$S=\frac{dFIR}{dT}=FIR(\frac{\Delta E}{kT^{2}})$$

**Figure 6:**
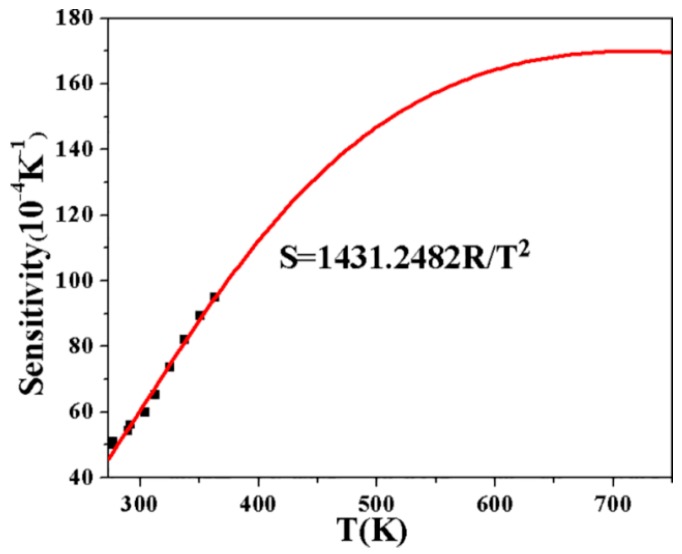
The experimental temperature sensitivity as a function of temperature ranging from 276 to 363 K.

The experimental temperature sensitivity as a function of temperature is presented Figure 6. Obviously, the maximum experimental sensitivity is 95 × 10^−4^ K^–1^ at 363 K. Table 2 lists the optical temperature sensing performance in different Er^3+^-doped host matrices. From the comparison, the newly-developed material demonstrates the highest sensitivity and may be a promising optical temperature sensing material.

## 4. Conclusions

A practically applicable optical fiber temperature sensor was designed and evaluated, based on the upconversion emission of Er^3+^/Yb^3+^ codoped TZNb glass. The basic physical properties of TZNb glass were investigated and the corresponding results demonstrated that TZNb glass possessed a good glass stability and high glass transition temperature, which was very advantageous to enlarge the work temperature range of optical fiber temperature sensor. Owing to the low phonon energy of TZNb glass, strong green upconversion emission was observed at a power of 1mW and the performance of temperature sensing was investigated using the fluorescence intensity ratio technique in which two thermally coupled levels of Er^3+^ ions was adopted. The maximum experimental sensitivity is 95 × 10^−4^ K^–1^ at 363 K, which suggests this point temperature sensor is an excellent optical thermometer with a relatively high sensitivity. 

## Figures and Tables

**Table 1:** Basic physical parameters of Er^3+^/Yb^3+^ codoped TZNb glass

Physical Parameters	Refractive Index (at 632.8 nm)	Density (g/cm^3^)	*T_g_* (K)	*T_x_* (K)	Δ*T* (K)
Value	2.0329	5.082	683	875	188

**Table 2:** Optical temperature sensing performance in the different Er^3+^ doped host matrices. The dopant, excitation power, maximum sensitivity, and the corresponding temperature are included.

Materials	Dopant	Excitation Power	Maximum Sensitivity (K^‒1^)	Temperature (K)	References
Lead germinate glass	Er-Yb	/	70 × 10^−4^	550	[5]
Fluorotellurite glass	Er	5 W/mm^2^	79 × 10^−4^	541	[11]
Tungsten–tellurite glass	Er-Yb	108 mW	28 × 10^−4^	690	[12]
Tellurite glass	Er-Yb	0.3 mW	39 × 10^−4^	/	[13]
Al_2_O_3_	Er-Yb-Mo	2 mW	51 × 10^−4^	443	[20]
Fluorophosphate glass	Er	/	54 × 10^−4^	630	[21]
Yb_2_TiO_7_	Er-Mo	5 mW	74 × 10^−4^	340	[22]
CaWO_4_ phosphor	Er-Yb	150 mW	73 × 10^−4^	518	[23]
β-NaLuF_4_	Er-Yb	/	52 × 10^−4^	303	[24]
TZNb glass	Er-Yb	1 mW	95 × 10^−4^	363	This work
